# Heavy Metal Contamination in the Aquatic Ecosystem: Toxicity and Its Remediation Using Eco-Friendly Approaches

**DOI:** 10.3390/toxics11020147

**Published:** 2023-02-03

**Authors:** Veer Singh, Nidhi Singh, Sachchida Nand Rai, Ashish Kumar, Anurag Kumar Singh, Mohan P. Singh, Ansuman Sahoo, Shashank Shekhar, Emanuel Vamanu, Vishal Mishra

**Affiliations:** 1 Department of Biochemistry, Rajendra Memorial Research Institute of Medical Sciences, Patna 800007, India; 2 School of Biochemical Engineering, Indian Institute of Technology, Banaras Hindu University, Varanasi 221005, India; 3 Centre of Bioinformatics, University of Allahabad, Prayagraj 211002, India; 4 Centre of Biotechnology, University of Allahabad, Prayagraj 211002, India; 5 Centre of Experimental Medicine & Surgery, Institute of Medical Sciences, Banaras Hindu University, Varanasi 221005, India; 6 Department of Botany, Banaras Hindu University, Varanasi 221005, India; 7 Udai Pratap Autonomous College, Varanasi 221002, India; 8 Faculty of Biotechnology, University of Agricultural Sciences and Veterinary Medicine of Bucharest, Bucharest 011464, Romania

**Keywords:** heavy metals, toxicity, source of heavy metal ions, eco-friendly, heavy metal removal

## Abstract

Urbanization and industrialization are responsible for environmental contamination in the air, water, and soil. These activities also generate large amounts of heavy metal ions in the environment, and these contaminants cause various types of health issues in humans and other animals. Hexavalent chromium, lead, and cadmium are toxic heavy metal ions that come into the environment through several industrial processes, such as tanning, electroplating, coal mining, agricultural activities, the steel industry, and chrome plating. Several physical and chemical methods are generally used for the heavy metal decontamination of wastewater. These methods have some disadvantages, including the generation of secondary toxic sludge and high operational costs. Hence, there is a need to develop a cost-effective and eco-friendly method for the removal of heavy metal ions from polluted areas. Biological methods are generally considered eco-friendly and cost-effective. This review focuses on heavy metal contamination, its toxicity, and eco-friendly approaches for the removal of heavy metals from contaminated sites.

## 1. Introduction

Environmental contaminants are substances that are present in the natural environment at levels higher than their permissible limits [1]. Industrialization and urbanization have emerged as the major causes of environmental pollution over the last few decades. The utilization of natural resources at a careless rate creates disturbances in the environment and causes several related problems [2]. There are several types of pollutants, such as organic, inorganic, metallic, gaseous and biological pollutants, which contaminate the environment [3]. Contamination from metallic ions in water arises due to several natural and anthropogenic activities, which harm animals and plants [4].

Heavy metals are characterized by their higher atomic weight or higher density. The term ‘heavy metal’ is used to describe metalloids or metallic elements which have toxic effects on humans and other living organisms [5]. Heavy metals including arsenic (As), chromium (Cr), lead (Pb), and cadmium (Cd) are toxic to humans, but a few heavy metals are not toxic; these include gold (Au) [6]. The density of heavy metals is commonly greater than 5 g/cm^3^: for example, Cr, Cd, and Pb [7]. The human body is generally exposed to heavy metal ions in one of four ways: the ingestion of metal-contaminated food, drinking contaminated water, skin contact, and inhalation in metal-contaminated air [8]. Metal compounds tend to form covalent bonds, which are responsible for the extremely toxic nature of metalloid compounds. Heavy metals can become covalently attached to organic groups and form lipophilic compounds or ions [9]. Due to the lipophilic nature of these metallic compounds, they pass through the cell membrane and enter the cell. These metallic compounds cause toxic effects when they interact with cell organelles [10].

Various technologies are available for the minimization of toxic metal ions from water. Physiochemical methods, such as filtration, ion exchange, reverse osmosis, precipitation, and physical adsorption, are frequently used for heavy metal treatment [11]. Precipitation is a well-known technique for the removal of Cr (VI), as well as Pb (II) and Cd (II). The precipitation is undertaken by varying the pH of wastewater [12,13]. These physiochemical techniques are expensive and generate secondary chemical sludge; moreover, these methods are only effective when the concentration of heavy metals is higher in the water (above 2 mM) [14]. Considering the disadvantages of these physiochemical methods, there is an urgent need to develop cost-effective and eco-friendly methods for the successful removal of heavy metals from water [15].

Bioremediation methods, such as biosorption, bio-reduction, bioaccumulation, mycoremediation, bacterial bioremediation, and phytoremediation, are considered inexpensive and eco-friendly methods [16]. Biosorption is an eco-friendly and inexpensive method which is used for the extraction of metal ions from contaminated water [17]. A variety of biomaterials, including rice and wheat husks, activated carbon, lignite, agro-waste, banana and *citrus limetta* peels, and green synthesized nanoparticles, are active biosorbents that can be used for the extraction of toxic metallic pollutants [18]. Living microbes, such as bacteria and fungi, are also effective remediating agents. This is due to the high metal-tolerance properties of fungi (*Pleurotus florida*) and bacteria. Several functional groups on the bacterial surface participate in the binding of heavy metals to the bacterial surface [19]. Heavy metals enter the cell and either bind to the cell membrane or are reduced into a less toxic form. The antioxidant systems of microbial cells reduce toxicity and aid bioaccumulation [20].

This review focuses on the sources of heavy metal contamination in water and its toxic effect on human beings and other living organisms. In this study, we also outline advanced, effective methods for the removal of heavy metals.

## 2. The Source and Toxicity of Heavy Metal Ions

Several industrial processes, including leather tanning, chrome plating, battery manufacturing, the glass industries, agricultural activities, domestic waste, and pharmaceutical industrial processes, are considered major sources of heavy metals, which generate toxic metal ions in the environment [21,22]. According to the International Leadership Association, about ten million tons of Pb (II) have been generated; 85.10% of the total amount of Pb (II) produced has been used in the battery industry, 5.5% in pigments, and the rest in the miscellaneous category (2.1%) (ILA, 2017). Pb (II) is also used in petrol as tetraethyl and tetramethyl agents in the form of antiknocking compounds [23]. Cr (VI) is used for steel production, wood preservation, chrome plating, pigments, and electroplating [24,25]. Cd is generally used in the electroplating and battery industries [26]. The Central Pollution Control Board (CPCB) has demarcated the maximum discharge limit of heavy metals in industrial effluent (Table 1).

The USA’s environmental protection agency (USEPA) has demarcated the maximum permissible limit of heavy metals in drinking water. The allowable concentrations of heavy metals in drinking water are listed in Table 2.

**Figure 1:**
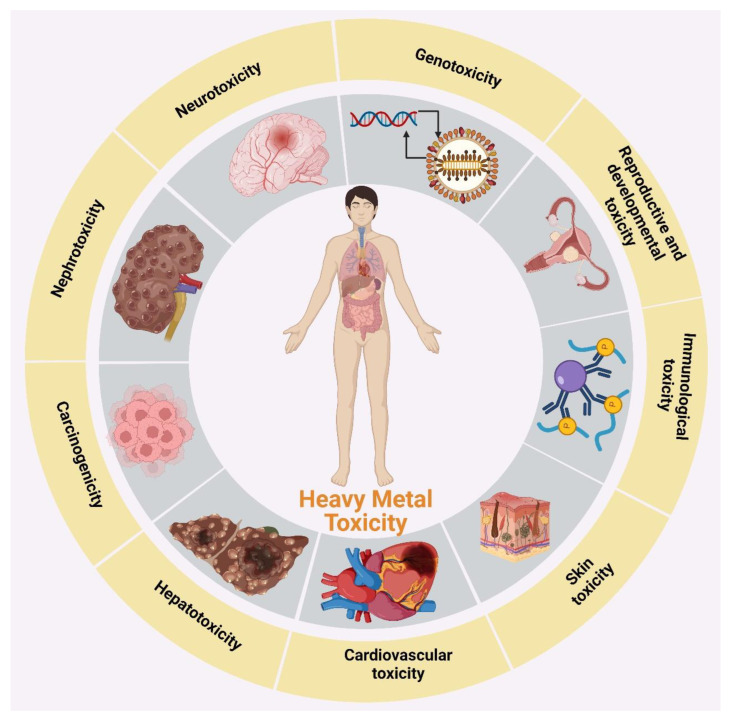
Toxicity of heavy metal ions.

Beyond these permissible limits, heavy metals cause several toxic effects. Some heavy metals are considered potential carcinogens [30,31]. The toxicity of heavy metal ions is shown in Figure 1.

Cr (VI) is a highly dangerous metallic ionic species, and its toxicity depends on its oxidation state [32,33]. It is a powerful oxidizing agent and shows much higher levels of toxicity than Cr (III). Cr (VI) enters the cell through the cell membrane and transforms into Cr (III) [34]. Cr (VI) reduces into Cr (III) and generates reactive oxygen species (ROS), which can damage internal components of the cell [35]. The Cr (VI) transformation process is known as a detoxification mechanism, which takes place away from the nucleus and other cell components. The reduction process also sometimes occurs outside of the cell through extracellular secretion [36]. Cr (VI)-reduction-mediated ROS generation can cause mutations in the DNA and damage cell organelles [37]. If the reduction of Cr (VI) occurs outside of the cell, Cr (III) is reduced and other intermediate reactions cannot deliver it into the cell, and toxicity is not observed [38]. Several researchers have noted that Cr (VI) is responsible for cancer and the damage of multiple organs, such as the liver and kidneys [39]. Gumbleton and Nicholls [40] indicated that Cr (VI) toxicity is responsible for kidney failure in rats. It has been reported that Cr (VI) is also responsible for respiratory cancer, the breakage of DNA strands, and abnormalities in chromosomes [41].

The main cause of Cd (II) exposure in humans is inhalation, through the ingestion of food and drinking of Cd (II)-contaminated water [42]. The chronic inhalation of Cd (II) causes a change in pulmonary functions, a reduction in olfactory functions, and emphysema [43]. Ingested Cd (II) causes abdominal pain, loss of consciousness, vomiting, nausea, hepatic injury, renal failure, gastrointestinal erosion, and a burning sensation [44]. It is also responsible for pulmonary adenocarcinomas and damage to single DNA strands, and disrupts the synthesis of proteins and nucleic acids [45].

Both natural and anthropogenic activities, such as mining, the burning of fossil fuels, and the manufacturing of batteries and glasses, are major sources of Pb (II) (https://fas.org/sgp/crs/misc/R46420.pdf (accessed on 16 June 2020). The toxic effects of Pb (II) in children come from dust and the chips used in packed food products, as it is used as a coating on the interior surfaces of packing materials [46]. The organs in the body that are most affected by Pb (II) toxicity are the kidneys, liver, and other soft tissues, such as the brain and heart [47]. Pb (II) toxicity massively impacts the nervous system. Poor attention, headaches, dullness, irritability, and memory loss are the early symptoms of Pb (II) poisoning in the central nervous system [48,49].

## 3. Removal of Heavy Metal Ions

The biological removal of heavy metals is more appealing than other conventional methods, because biological methods are cost-effective, eco-friendly, and efficient when removing low concentrations of heavy metal ions from wastewater [12]. Several biological agents, such as plant biomass, agricultural waste, microbial biomass, green synthesized nanoparticles, fruit waste, and biopolymers, have been utilized for the removal of heavy metals [50,51]. Living organisms, such as bacteria, algae, and fungi, also increasingly play a role in the removal of heavy metals [52]. Several microbes, such as fungi, bacteria, and algae, take heavy metals into their cells from the surrounding medium [53]. It has been reported that various microbial species can transform highly toxic Cr (VI) into less toxic Cr (III). Microorganisms can easily take Cr (III) into their cells as it has lower solubility in water and low toxicological properties [54]. Several heavy metal bioremediation methods, including biosorption (using dead biomass), phytoremediation (plant-mediated heavy metal removal), bioreduction (the conversion of oxidation states of heavy metal ions), and bioaccumulation (the uptake of heavy metal ions into intracellular space) have been proposed in past [55]. Heavy metals can be remediated using metabolically independent (dead materials) and metabolically dependent (live cells of bacteria, fungi, and algae) agents.

### 3.1. Metabolically Independent Approaches for Heavy Metal Removal

The phenomenon of biosorption is considered to be metabolically independent, and the biosorption process is generally performed by dead biomass, such as plant biomass, agro-waste, microbial biomass, and green synthesized nanomaterials. In biosorption, heavy metal ions bind to the surface of the adsorbent through several functional groups that are present on the surface [18,56]. However, it is also performed by live cell biomass; living microbial cells can bind heavy metal ions on their cell surface by passive adsorption and complexation [57,58].

Biosorption can be described as the binding of heavy metal ions to surface functional groups, including amino, amide, imidazole, sulfonate, and carboxyl groups [59]. The pKa value of the medium also plays important role in the adsorption of heavy metal ions and the binding tendencies of functional groups [60]. One of the most important and memorable aspects of biosorption is the selection of a suitable biosorbent. The raw material used for adsorption should be easily available, non-toxic, and cost-effective [61]. The dispersity of surface groups also determines the biosorbent‘s quality. For an ideal biosorbent, the density of the surface functional groups should be high [62]. The surface morphology is an important characteristic of adsorbents, and plays an important role in heavy metal adsorption. Rough and porous surfaces provide a larger surface area, and are advantageous for binding heavy metal ions on the biosorbent’s surface [63]. It is important to characterize the surface morphology and functional groups of biosorbents. Various techniques, such as Fourier transformation infra-red (FTIR), scanning electron microscopy (SEM), energy dispersive X-ray analysis (EDX), nuclear magnetic resonance (NMR), and X-ray diffraction (XRD), are used for the characterization of biosorbents [64,65].

Biosorption is affected by several factors including the morphology and types of biomass, the presence of more than one metal ion in the media, and the surrounding temperature and pH of the medium [66]. Commonly, a decrease in the pH is responsible for competition between cationic heavy metal ions. However, elevation in pH is responsible for the deprotonation of the adsorbent surface and causes the exposure of surface functional groups [66,67]. The regeneration of the biosorbent can be also achieved by using desorption. The recovery of metal ions was achieved by varying the pH of the medium [17,68]. In addition, the Cr (VI) biosorption mechanism is a complex phenomenon in which a Cr (VI) ion is attached to the biosorbent’s surface and reduced into less toxic Cr (III) [69].

The reduction-cum-adsorption of Cr (VI) usually occurs in three stages: (1) the binding of negatively charged chromate ions to the positive functional groups; (2) the reduction of Cr (VI) into Cr (III) through an electron donated by the functional groups; (3) the release of Cr (III) ions into the solution, with the remaining Cr (III) ions staying bound to the adsorbent surface with negatively charged surface functional groups [70,71,72]. 

The biosorption phenomenon can be also performed on immobilized biomass, such as bacterial biomass on a solid surface, fungal biomass on a supportive medium, or dead biomass immobilized on another supportive surface. Immobilization improves the rigidity, strength, and age of the biosorbent and improves its biosorption capacity [73]. Various types of matrices are available and are used for immobilization; these include alginate and polyurethane [74,75]. Additionally, several microbial and plant biomasses have been utilized for immobilization on the above-described matrices: for example, the biomass of *Chlorella homosphaera* immobilized on a sodium alginate matrix [76]. Table 3, Table 4 and Table 5 show the uptake capacity of various biosorbents in terms of Cr (VI), Cd (II), and Pb (II), respectively.

The above-mentioned biomasses are considered effective biosorbents for the removal of heavy metal ions. In addition, nanoparticles are receiving increased attention in the field of heavy metal removal. Nanoparticles also have applications in electron devices, health care, energy, agriculture, and wastewater treatment [94,95]. In general, nanoparticles are small in size within the nanoscale (1–100 nm), and the small size of nanomaterials provides a large surface area as compared to bulk materials [96]. Along with their small particle size, nanoparticles have some other important properties, such as quantum and macro-quantum tunnel effects [94,96]. These specific properties of nanoparticles are responsible for their unusual reactivity and adsorption properties. These properties of nanomaterials are favorable for the biosorption of heavy metals from contaminated media [94,97]. Several types of nanoparticles have been synthesized for the removal of heavy metal ions, and a few examples of these nanomaterials and their heavy metal adsorption capacities are shown in Table 6.

### 3.2. Metabolically Dependent Approaches for Heavy Metal Removal

Bioaccumulation is a phenomenon in which heavy metal ions accumulate inside the microbial cells [106,107]. It is a highly complex phenomenon and slower than biosorption, because several metabolic pathways actively participate in this process [106]. The bioaccumulation process is performed by living microbial cells in the suspension medium and requires continuous observation [108]. It minimizes several steps of biosorbent preparation, such as the harvesting of biomass, the drying of biomass, and the preparation (washing and crushing) and storage of biomass [109]. However, bioaccumulation is hypersensitive to experimental conditions [110]. Pollutants present in wastewater affect microbial growth and also cause competition with the heavy metal ions selected for bioaccumulation. Moreover, these pollutants can bind to the bacterial outer surface and disrupt heavy metal accumulation in cells [111]. Several fungi, bacteria, algae, and plant species have been shown to play an important role in the removal of heavy metals.

**Figure 2:**
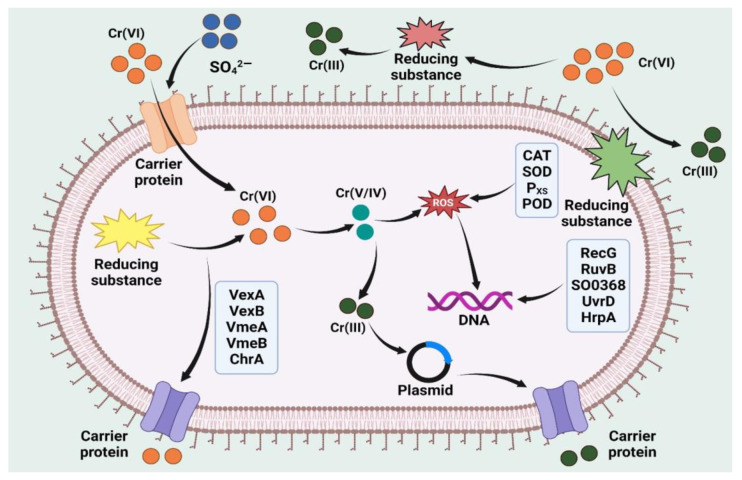
Bacterial-mediated Cr (VI) remediation and the mechanism of Cr (VI) reduction in the bacterial cell.

Among these living systems, microbes have a good ability to remove heavy metals, and also possess resistant properties. Bacteria can uptake heavy metal ions through cell surface receptors and accumulate heavy metal ions inside the bacteria [112]. Several bacterial cell, such as those of the *Pseudomonas* species [113], the *Klebsiella* species [114], and the *Microbacterium* species [115], are resistant to the heavy metal ions isolated from several contaminated sites [116]. Bacteria isolated from heavy-metal-contaminated sites generally became resistant to heavy metal ions [117]. Several researchers have identified heavy-metal-resistant *Microbacterium* strains from contaminated areas. Henson et al. [118] isolated a *Microbacterium* sp. (Cr-K29) that was capable of removing 88% of Cr (VI) from contaminated water. According to Pattanapipitpaisal et al. [119] *Microbacterium liquefaciens* is capable of removing 81% of Cr (VI) from wastewater in an immobilized microbial form on a solid surface. Bacteria utilize various mechanisms for the removal of heavy metals. Bacteria can utilized heavy metal ions for their metabolic activity, or else they detoxify heavy metal ions through soluble enzymes produced by the bacterial cells [120]. The overall bioremediation of Cr (VI) by bacteria is shown in Figure 2.

ROS are generated in bacterial cells when these cells are exposed to toxic heavy metals. ROS damage the cell organelles and affect several metabolic functions which control normal cell functions [121,122]. Thus, antioxidant systems, such as superoxide dismutase (SOD) and catalase in the microbial cells, are actively involved in the detoxification of ROS-generated stress in bacterial cells [123]. Several heavy-metal-resistant bacterial species have been identified by researchers. The Cr (VI), Pb (II), and Cd (II) bioremediation efficiency of heavy-metal-tolerant bacterial strains is shown in Table 7, Table 8 and Table 9, respectively.

### 3.3. Other Bio-Remedial Techniques

Other bio-remedial techniques, such as phytoremediation, capacitive deionization, electrosorption, the metagenomic approach, and diatom-mediated heavy metal bioremediation, can effectively be used in wastewater treatment.

Phytoremediation is a plant-based technique used for the removal of heavy metal ions and other contaminants from water and soil. Plants can tolerate heavy metal stress and can grow in areas highly contaminated with heavy metals. Hence, plants can remove heavy metals from water and soil by taking up heavy metals through their roots. There are some disadvantages to phytoremediation; some plants require large areas to grow and have long growth times, and it is difficult to maintain the growth of some plants in water [134].

The capacitive deionization (CDI) process has gained attention as a possible method for heavy metal removal in recent years. The cost-effective and widely available materials have facilitated the growth of electrosorption research. Hence, CDI is based on inexpensive and eco-friendly methods for the removal of heavy metals from wastewater. CDI has recently shifted towards electrosorption for the removal of selective heavy metal ions from water, and it is suitable for industrial wastewater treatment [135].

Metagenomics is a technique used for the identification of microbial communities in environmental samples. Several cultured and uncultured microbes are present in the soil, water, and air. Some of these microbes can grow in high-stress conditions, such as heavy metal stress. These microbes are resistant to heavy metal ions and can take up heavy metals into their intracellular space, or convert them into less toxic forms [136].

Heavy metal ions are present in both freshwater and marine ecosystems. Diatoms play an important role in the removal, degradation, and detoxification of heavy metal ions from contaminated sites [137]. Diatom algae are diverse and have extensive evolutionary variations in their metabolic pathways. Diatoms show higher productivity and can easily grow in an aqueous medium. Diatoms utilize abiotic and biotic mechanisms to contaminate and easily adapt to varying environmental conditions. Diatom-based strategies are considered a cost-effective and sustainable method for the removal of heavy metal ions from contaminated water [138].

### 3.4. Disadvantages and Advantages of Methods of Heavy Metal Removal

Metabolically dependent heavy metal removal is required for growth-specific media used for microbial growth. Moreover, other growth parameters, such as pH, temperature, and agitation, are necessary for the proper growth of microbes [97]. Hence, bioremediation methods based on living microorganisms require more energy input and other media components. Heavy metal removal methods based on non-living organisms are metabolically independent and do not require media or a great deal of energy input, such as that required by living organisms. Moreover, biological methods are yet to be established and commercialized. Beyond these disadvantages, these biological methods are cost-effective, eco-friendly, and effective at very low concentrations of heavy metal ions in water [97,139].

### 3.5. Challenges of Heavy Metal Remediation Technologies and Future Prospects

Heavy metal remediation technologies are time-consuming and laborious. In addition, the major challenges for bioremediation techniques concern their implementation at the commercial level and their application in water treatment plants [140]. These techniques involve the use of chelating agents and surfactants for the modification of the adsorbent surface, and bacterial immobilization on the solid surface and the use of composite nanomaterial or biopolymers to increase the removal of heavy metals from water. However, future research can explore the potential of using biological remediation methods for the removal of other toxic metal ions such as As, Ni, Hg, and Cu. Advanced technical duty/assessment tools, such as lifecycle assessments and the exergo-environmental and exergo-economic investigation of large-scale wastewater treatment plants, can also be used in future to develop cost-effective, energy-saving, and eco-friendly systems. Additionally, the molecular mechanisms of heavy metal removal and comparative transcriptomic analysis may be considered in future research [12,34].

## 4. Conclusions

Heavy metal ions are used in many industrial processes, such as tanning and chrome plating. Heavy metal ions including Cd (II), Pb (II), As (III and V), and Cr (VI) are the substances that pose the most danger to human health. Some of these metal ions are also released into the environment and cause contamination. The presence of heavy metal ions in the water and soil affects human health, as well as the health of some aquatic and terrestrial animals. Several types of cancer, kidney and liver damage, skin problems, and mental dullness are well-known health issues caused by heavy metal toxicity. Membrane filtration, reverse osmosis, chemical reduction, and adsorption are the major conventional methods used for the removal of heavy metal ions from wastewater [141]. However, these methods generate large amounts of secondary pollutants and are costly compared to biological methods. These eco-friendly methods have many more advantages than conventional methods. In this review, we summarized the biological methods, including both metabolically dependent and metabolically independent methods, for heavy metal removal. Moreover, this review also focused on the sources of heavy metals and their toxicity. This study improves our understanding of how to design effective biological wastewater treatment systems.

## Figures and Tables

**Table 1:** Maximum dischargeable limits for heavy metals in industrial effluent [27].

Heavy Metals	CPCB, 2000 (Dischargeable Limit in Industrial Effluent)
Cr (VI)	1.00–2.00 mg/L
Cd (II)	0.20–2.00 mg/L
Pb (II)	0.10 mg/L

**Table 2:** The maximum allowable limit of heavy metal ions in drinking water [28,29].

Heavy Metals	US EPA, 2018	WHO, 2011
Cr (total) (mg/L)	0.10 mg/L	0.05
Cd (II) (mg/L)	0.005 mg/L	0.003
Pb (II) (mg/L)	0.015 mg/L	0.01

**Table 3:** Cr (VI) removal by microbial and plant-based biomasses, and Cr (VI) biosorption capacity.

Biosorbent	Biosorption Capacity (mg/g)	pH	Temperature (°C)	Biosorbent Dose (g/L)	Initial Concentration (mg/L)	Reference
***Trewia nudiflora*** **fruit peel powder**	294.12	1–2	20	0.75	22–248	[77]
***Ceramium virgatum*** **dry biomass**	26.5	1.5	20	10	10	[78]
**Pine needle powder**	48	2–3	25	10	50	[79]
***Dictyota dichotoma*** **biomass**	9.02	4	27	20	40	[80]
***Cupressus lusitanica*** **Bark**	305.4	1.5	28	10	100	[69]

**Table 4:** Cd (II) removal using microbial and plant-based biomass.

Biosorbent	Biosorption Capacity (mg/g)	pH	Temperature (°C)	Reference
Okara waste	14.80	6.2	70	[81]
Foxtail millet shell	12.48	5	25	[58]
Heat-inactivated marine *Aspergillus flavus*	174.25	7	20	[82]
*Morus alba* L. pomace	21.69	6	40	[83]
Pomelo fruit peel	13.35	5.5	30	[84]
Wheat straw biochar	69.80	5	25	[85]
*Klebsiella* sp. biomass	170.4	5	30	[86]
Extracellular Polymeric Substances (EPS) synthesized by microbactan	97	7	28	[87]

**Table 5:** Pb (II) biosorption capacity of different plant- and microbial-biomass-based biosorbents.

Biosorbent	Biosorption Capacity (mg/g)	pH	Temperature (°C)	Reference
Heat-inactivated marine *Aspergillus flavus*	207.2	6	20	[88]
Pomelo fruit peel	47.18	5.5	30	[84]
*Citrus grandis* peels	2.13	3	50	[89]
Pea (*Pisum sativum*) peels	140.84	6	30	[90]
Meranti sawdust	34.24	6	30	[91]
*Solanum melongena* leaves	71.42	5	40	[92]
*Araucaria heterophylla* (green plant) biomass	9.64	5	30	[93]

**Table 6:** Nanomaterials and their heavy metal adsorption capacity.

Nanoparticles/Nanocomposites	Heavy Metals	Biosorption Capacity (mg/g)	pH	Temperature (°C)	Reference
Chitosan-functionalized magnetic nanoparticles	Pb (II)	498.6	6	30	[98]
CuO nanostructures	Pb (II)	115–125	6.5	--	[99]
Cerium dioxide nanoparticles (CeO_2_ NPs)	Pb (II)	4.99	6.8	30	[100]
Iron oxide–tea waste nanocomposite	Pb (II)	18.83	--	25	[101]
Nanoscale zerovalent iron (nZVI)	Pb (II)	1667	4.5	35	[102]
Carboxymethyl cellulose bridged chlorapatite nanoparticles	Cd (II)	150.2	7	--	[103]
Alumina nanoparticles	Cd (II)	24.20	8	27	[104]
CNSR-coated magnetic nanoparticles	Cd (II)	54.6	10	30–50	[105]
Oxide–silica composite	Cd (II)	43.45	6	50	[88]

**Table 7:** Bacterial species and their Cr (VI) removal efficiency.

Bacteria	Removal Efficiency (%)	Optimum pH	Optimum Temperature	Initial Cr (VI) Concentration (mg/L)	Reference
***Staphylococcus capitis***	89	7	37	--	[124]
***Bacillus*** **sp. JDM-2-1**	86	6	37	--	[124]
***Bacillus subtilis*** **(Bacteria)**	95.19	7	37	--	[125]
***Acinetobacter*** **sp.**	75	7	75	--	[126]

**Table 8:** Pb (II) removal efficiency of bacterial strains.

Bacteria	Removal Efficiency (%)	Optimum pH	Optimum Temperature	Initial Pb(II) Concentration (mg/L)	Reference
*Oceanobacillus profundus* KBZ 3-2	97	6	30	50	[127]
*Acinetobacter* sp. strain THKPS16	71.2	5	35	35	[128]
*Citrobacter* sp. Strain MKH2	95.06	-	30	80	[129]
*Bacillus* sp. Strain Q3	93.8	5.8	38.8	115.4	[130]
*Bacillus* sp. Strain Q3	76.4	6.2	34.3	127.4	[130]

**Table 9:** Cd (II) removal efficiency of bacterial strains.

Bacteria	Removal Efficiency (%)	Optimum pH	Optimum Temperature	Initial Cd (II) Concentration (mg/L)	Reference
*Bacillus* sp. Strain Q3	58	5	38.6	50.6	[130]
*Bacillus* sp. Strain Q3	78	5	38.3	50	[130]
*Cedecea* sp. strain SC19	51	7	37	120	[131]
*Stenotrophomonas maltophilia* ZZC-06	81.43%	6	30	10	[132]
*Pseudomonas azotoformans* strain JAW1	44.67	6	30	25	[133]

## Data Availability

Not applicable.
